# Treatment of Hydrocele

**Published:** 1893-07

**Authors:** 


TREATMENT OF HYDROCELE.—In a late issue of the *British Medical Journal*, Dr. Hall reports the successful treatment of nineteen cases of hydrocele by an extremely simple operation. The operation consists in slitting up the scrotum and tunica vaginalis, evacuation of the fluid, when the tunica vaginalis is sewed to the outer skin and a dressing of carbolized oil applied at a single sitting.—*Ex.*

